# Para-articular osteochondroma of the infrapatellar fat pad: A report of three cases

**DOI:** 10.1016/j.ijscr.2020.03.030

**Published:** 2020-04-03

**Authors:** Takahiro Nishimura, Isaku Saku, Shotaro Kanda, Takashi Fukushima, Toru Akiyama

**Affiliations:** Department of Orthopeadic Surgery, Saitama Medical Center, Jichi Medical University, Saitama, Japan

**Keywords:** Para-articular osteochondroma, Ossification, Infrapatellar fat pad, Simple marginal excision, Surgery, Knee

## Abstract

**Introduction:**

Para-articular osteochondromas are not very common. We performed simple marginal excision for three cases.

**Presentation of case:**

We encountered three cases of para-articular osteochondroma of the infrapatellar fat pad. All patients were men, with an average age of 43 years. The symptoms were very similar to those of anterior knee pain, which were increasing swelling and reduction of the range of motion. After simple marginal excision was performed, the symptoms improved and no recurrences or malignant transformation occurred during the mean postoperative period of 51 months.

**Discussion:**

Forty-seven cases of para-articular osteochondroma have been reported, and excision is the standard treatment. Local recurrence has been reported in only one case, and malignant transformation has never been reported. However, in some reports there was no specification of the excision method, which was simply referred to as “excision.” Thus, we used a simple marginal excision for all three of our cases.

**Conclusion:**

We report three cases of para-articular osteochondroma of the infrapatellar fat pad that were effectively treated using simple marginal excision.

## Highlights

• Three cases of para-articular osteochondroma, a rare benign disease, are presented.
• Symptoms at presentation included chronic knee pain and limited knee movement.
• Simple marginal resection using an open approach was performed.
• Post-surgery, the pain disappeared and the range of motion of the knee was restored.

## 1 Introduction

Osteochondromas are common bone tumors that usually originate in the ends of bones. Most are benign, although a few become malignant. Osteochondromas not attached to a joint, such as those arising from the infrapatellar fat pad, are rare; they are termed para-articular osteochondromas and were first described by Jaffe in 1958 [1]. Para-articular osteochondromas are composed of bone and cartilage and arise in the fibrous joint capsule or the soft tissue. Similar diseases include synovial chondromatosis and soft tissue chondroma.

In the English literature, only 47 para-articular cases have been reported, including osteochondroma, chondroma and ossifying chondroma [[2], [3], [4]]. Of these 47 cases, 30 were finally pathologically diagnosed as osteochondroma. Most patients underwent excision; however, the surgical method was not unified. Furthermore, there were very few reports of several cases treated using the same excision method [2,5,6].

We performed open simple marginal resection for para-articular osteochondroma of the infrapatellar fat pad. Herein, we report the clinical outcome of our 3 cases. This work has been reported in line with the PROCESS criteria [14].

## 2 Presentation of case

All 3 patients in our study were men with an average age of 43 years (range, 35–55 years). All had a history of anterior knee pain and presented with slowly increasing swelling around the distal patella. The duration of the presenting symptoms was 100 months (range, 60–120 months). The range of motion of the affected knee was limited, and pain was most severe when the knee was fully flexed. In all three cases, the lesions arose from the infrapatellar fat pad.

For each patient, we performed open simple marginal resection because of the possibility of synovial osteochondromatosis or para-articular osteochondroma. The preoperative pain disappeared and the range of motion of the knee joint was restored in 38–72 months (average, 51 months) after surgery. There were no recurrences or malignant transformation (Table 1).

**Table 1:** Patient characteristics.

Case No.	Age	Sex	History	ROM		Follow-up	Recurrence
				Pre-operation	Post-operation		
1	35	M	120 months	−30–95°	Normal	54 months	None
2	38	M	60 months	−10–80°	Normal	26 months	None
3	55	M	120 months	0–125°	Normal	20 months	None

We describe one of the three cases in detail. A 35-year-old man presented with a 120-month history of swelling and pain in the anterior region of the right knee. He had no history of trauma. The pain was strongest when the knee was flexed. The swelling reduced the range of motion of the right knee: extension was −30 degrees and flexion was 95 degrees compared with 0 degrees and 130 degrees in the left knee, respectively. The right knee was stable. The mass was located in the infrapatellar pad and was hard and immovable, but not warm to the touch. The results of blood tests, including blood counts, were normal.

**Fig. 1:**
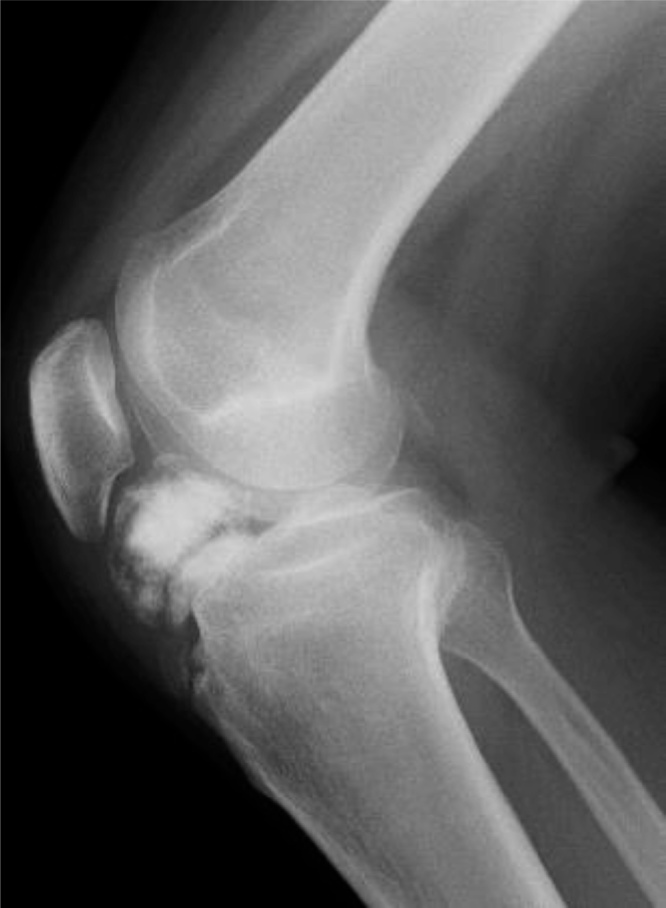
Plain radiography (lateral view) showed a finely circumscribed osseous lesion in the infrapatellar fat pad.

**Fig. 2:**
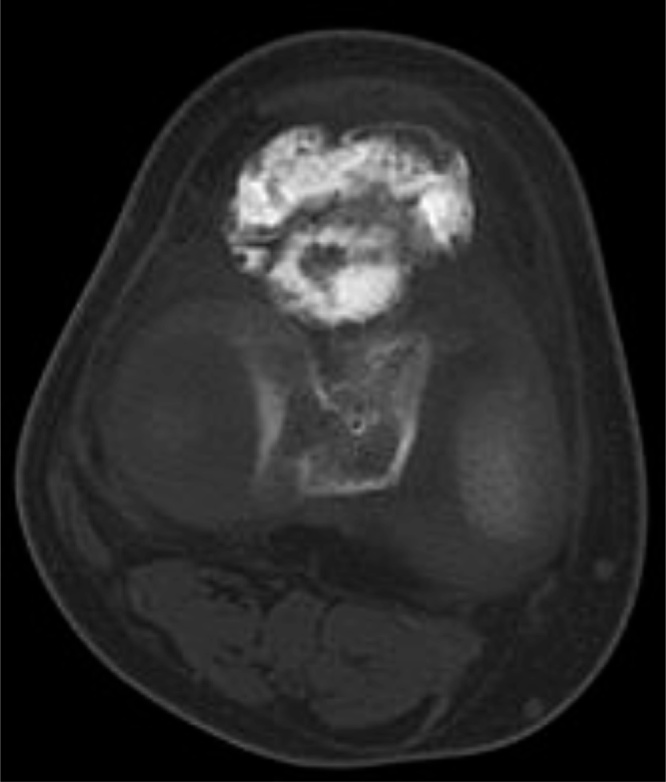
Computed tomography revealed an osseous lesion in the infrapatellar fat pad that was not attached to the bone or the articular cartilage.

**Fig. 3:**
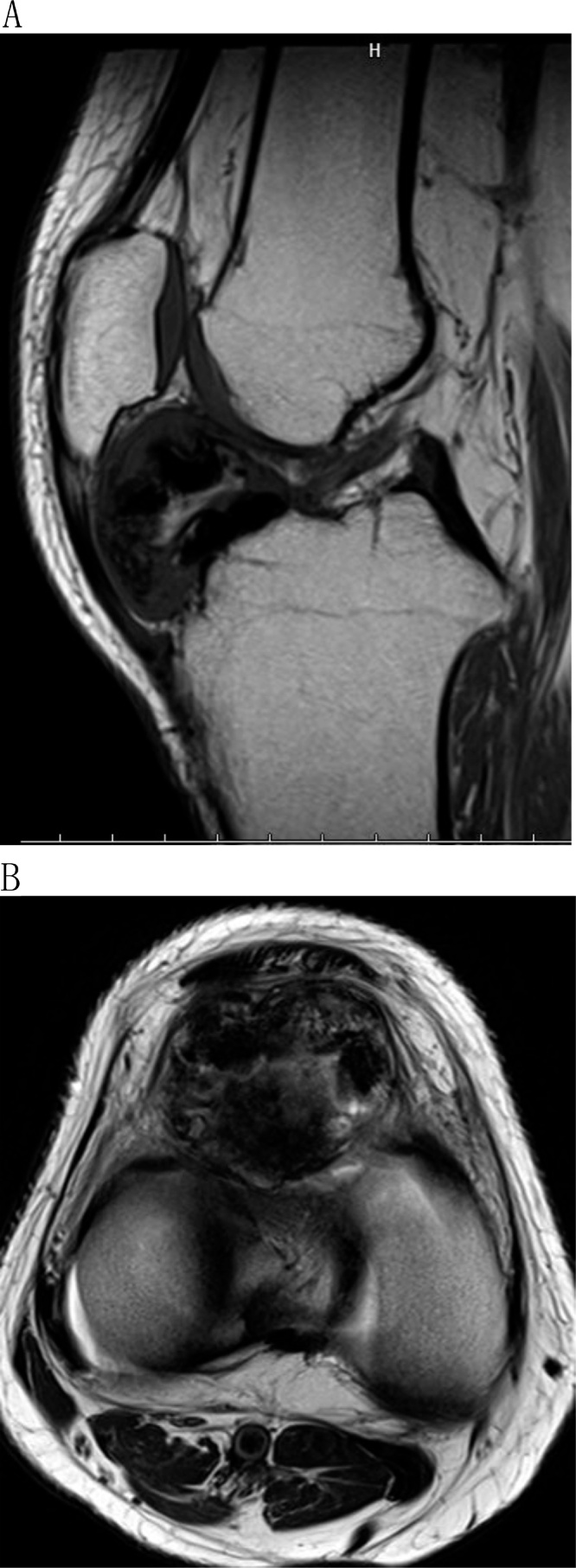
A: T1-weighted magnetic resonance imaging (MRI) showed low signal intensity in the infrapatellar fat pad that indicated ossification. B: T2-weighted MRI showed a high intensity area in the lesion that corresponded to cartilage.

Plain radiography (lateral view) showed a finely circumscribed osseous lesion in the infrapatellar fat pad (Fig. 1). Computed tomography revealed an osseous lesion in the infrapatellar fat pad that was not attached to the bone or the articular cartilage (Fig. 2). T1-weighted magnetic resonance imaging (MRI) showed low signal intensity in the infrapatellar fat pad that indicated ossification, and T2-weighted MRI showed a high intensity area in the lesion that corresponded to cartilage (Fig. 3A, B). Open simple marginal resection was performed using an medial para-patella approach. The mass in the infrapatellar fat pad was completely extra-articular and was not attached to the patella tendon.

**Fig. 4:**
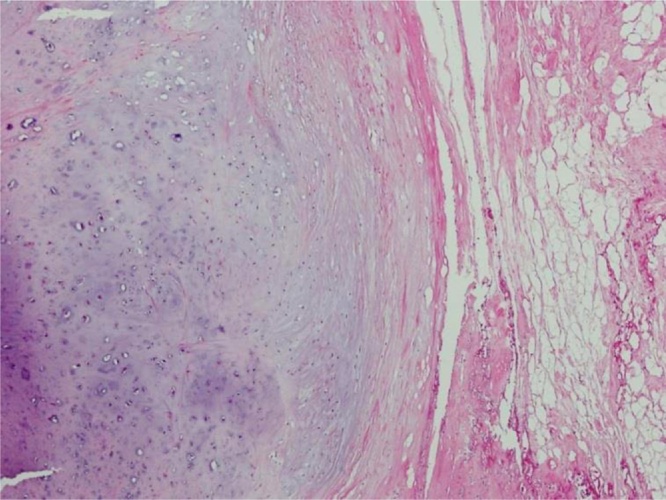
The mass included hyperplastic cartilage but no synovial tissue or atypical cells.

As shown in a photomicrograph, the main component of the resected mass was a hyperplasia cartilage matrix within the adipose tissue. The mass did not physically contact the synovial joint and did not contain any atypical cells (Fig. 4). Thus, the diagnosis was para-articular osteochondroma.

Two weeks after surgery, the patient’s pain disappeared, and the range of motion in the right knee was the same as that in the left knee. Moreover, the patient was able to sit up straight. There has been no local recurrence during the 72 months after surgery.

## 3 Discussion

Para-articular osteochondromas are benign lesions in the soft tissue surrounding the joints and consist of bone and cartilage. Thirty cases of pathologically diagnosed para-articular osteochondroma have been reported. Para-articular osteochondroma is differentiated from synovial chondromatosis by the absence of cartilaginous or osteocartilaginous nodules in the synovium, which may result in untethered bodies in the joint cavity [1].

Para-articular osteochondroma has no sex predilection. Its major symptoms are joint or mass swelling, anterior knee pain, and decreased range of motion of the knee. Most patients present with a several-year history of these symptoms, with the mass growing slowly. Pain was the main presenting symptom in 73% of cases, while a mass occurred in 91% of cases. The mean duration of presenting symptoms was 63.9 months. Our 3 patients’ presenting symptoms were pain and mass swelling, and the symptom duration was 100 months [2].

The cause of para-articular osteochondroma remains unknown. Several reports have associated it with minor injuries [[7], [8], [9]]. Evaniew et al. [2] reported that only 16% of cases involved antecedent trauma. The patients in our report had no history of minor or major injuries. Some reports have also suggested that ossification of the infrapatellar fat pad represents the end stage of Hoffa’s disease [[10], [11], [12]].

Para-articular osteochondroma histologically differs from synovial osteochondromatosis in two respects: it has no malignant features, and the endochondral ossification appears as an interface between the bone and cartilage, with no synovial tissue in the tumor. As reported by Sakai et al. [5], para-articular osteochondromas mainly consist of bony tissue with a relatively small amount of cartilaginous tissue. They also contain partially ossified cartilage, and the cartilaginous lobular tissue is separate from the connective tissue [5]. These histological features allow us to distinguish para-articular osteochondroma from various similar osteochondral lesions. In the patients in our report, the masses included hyperplastic cartilage but no synovial tissue or atypical cells.

Open excision is the standard treatment for para-articular osteochondroma. Excision with intralesional arthroscopic technique has been reported in two cases [4,13]. Local recurrence after excision has been reported in only one case [10], and malignant transformation has never been reported. We performed simple marginal resection in our 3 cases, as did Ogura et al. [9]. For our 3 cases, the average postoperative follow-up was 51 months (38–72 months), which was longer than the mean 14.9 months in a previous report [2]. There were no complications, such as infection, neurovascular injury, or wound breakdown, nor were there any recurrences or malignant transformation.

## 4 Conclusion

We report three cases of para-articular osteochondroma of the infrapatellar fat pad, all of which were effectively treated using simple marginal excision. The postoperative course was uneventful; pain disappeared and normal range of motion was restored. There was no local recurrence or malignant transformation during the average follow-up period of 51 months.

## Declaration of Competing Interest

The authors declare that there is no conflict of interests regarding the publication of this paper.

## Sources of funding

Authors declare there are no funding resources for this paper.

## Ethical approval

Institutional review board approval was not required because all data were collected from clinical records and imaging systems for routine preoperative planning and follow-up.

## Consent

Written informed consent was obtained from all of the patients for publication of this case report and accompanying images. A copy of the written consent is available for review by the Editor-in-Chief of this journal on request.

## Author contribution

TN wrote this paper. IS and TA attended the surgery, and all authors read this paper.

## Registration of research studies

We have registered our research at http://www.researchregistry.com.

The UIN of our study is “researchregistry5380”.

## Guarantor

Isaku Saku is the corresponding author of this paper.

## Provenance and peer review

Not commissioned, externally peer-reviewed.

## Patient perspective

The patient shared her perspective on the treatment when her wound was healed completely.

## CRediT authorship contribution statement

**Takahiro Nishimura:** Data curation, Writing - original draft. **Isaku Saku:** Conceptualization, Methodology, Writing - review & editing. **Shotaro Kanda:** Visualization. **Takashi Fukushima:** Visualization. **Toru Akiyama:** Supervision, Project administration.
